# Absence of Evidence or Evidence of Absence? Commentary: Captured by the pain: Pain steady-state evoked potentials are not modulated by selective spatial attention

**DOI:** 10.3389/fnhum.2016.00252

**Published:** 2016-06-14

**Authors:** Elisabeth Colon, André Mouraux

**Affiliations:** ^1^Institute of Neuroscience, Université Catholique de LouvainLouvain-la-Neuve, Belgium; ^2^Center for Pain and the Brain, Department of Anesthesiology, Perioperative and Pain Medicine, Boston Children's Hospital, Harvard Medical SchoolBoston, MA, USA

**Keywords:** pain, selective attention, spatial attention, top-down attention, EEG, neurophysiology, human neurosciences, painful stimulation

A broad range of cognitive factors modulates behavioral, and cortical responses to painful stimuli, and pain perception. Among them, attention plays an important role (Wiech et al., 2008). Selective attention to a sensory modality or selective spatial attention to a given body part can modulate the processing of painful stimuli (Spence et al., 2002; Legrain et al., 2012). Painful stimuli also yield the ability to capture involuntary attentional processes depending on their salience and relevance for current goals (Legrain et al., 2012). Yet, previous electrophysiological studies that have assessed the effect of selective attention on pain have mostly used very short and transient stimuli (Legrain et al., 2012). Recently, Blöchl et al. (2015) used steady-state evoked brain potentials (SS-EPs) to investigate the effect of selective spatial attention on the cerebral processing of sustained painful stimuli. SS-EPs reflects a sustained cortical response induced by the periodic modulation of a long-lasting stream of sensory input (Regan, 1989). Using this approach, top-down effects of attention on the cortical processing of intramodal sensory inputs have been demonstrated in visual, auditory, and somatosensory modalities (Morgan et al., 1996; Giabbiconi et al., 2004, 2007; Bidet-Caulet et al., 2007). Typically, selectively attending to one of several concurrently presented streams of sensory inputs increases the magnitude of the SS-EP elicited by the attended stream. Blöchl and collaborators hypothesized that selectively attending to one of two painful inputs applied on the hands would lead to a selective enhancement of the magnitude of the SS-EP elicited by the attended input. Unlike their prediction, they failed to demonstrate such a modulation. They argued that attention cannot be effectively shifted between two simultaneously applied sustained painful stimuli, and that this would constitute a unique property of pain as compared to other senses. Although, we understand their interpretation, in our opinion, their results do not fully justify this interpretation.

A first concern is that other studies have failed to demonstrate top-down attentional modulation of SS-EPs elicited by two sensory inputs belonging to the same sensory modality. In a pioneering EEG study, Linden et al. (1987) found no evidence of an attentional modulation of auditory SS-EPs whereas more recent research found an effect and suggested that the attentional modulation may depend on the experimental context (Müller et al., 2009). Attentional modulation of innocuous somatosensory SS-EPs may also depend on the modulation frequency, the task difficulty, or the experimental design (Adler et al., 2009; Katus et al., 2014). Taken together, this suggests that top-down attentional modulation of SS-EPs is highly context-dependent. Therefore, the lack of effect reported by Blöchl and collaborators could result from the specific experimental context of their study, rather than to the fact that the eliciting stimuli were painful.

Accordingly, the modulation frequencies used by Blöchl and collaborators (31 and 37 Hz) are quite different from those usually used to elicit somatosensory SS-EPs. Tobimatsu et al. (1999) found that the optimal frequency range to elicit non-nociceptive somatosensory SS-EPs lies between 20 and 30 Hz with a maximum around 21 Hz. Moreover, attentional modulation of non-nociceptive somatosensory SS-EPs has been mostly reported using modulation frequencies between 20 and 26 Hz (Giabbiconi et al., 2004, 2007), and intermodal attentional modulation of nociceptive SS-EP has been shown at 6 Hz (Colon et al., 2014). Furthermore, using similar frequencies (30 and 34 Hz) and a simple detection task, Adler et al. (2009) failed to demonstrate attentional modulation of non-nociceptive somatosensory SS-EPs. However, when slightly decreasing the modulation frequencies (28 and 30 Hz) and using a more demanding discriminative task, they observed a significant effect. Consequently, both the modulation frequency and the task may be critical to observe top-down attentional modulation of SS-EPs.

Another methodological difference concerns the timing of the cue that defined the attended stream in Blöchl and collaborators. Their cue occurred 3 s *after* the onset of the stimulation trains, whereas in most previous studies in the somatosensory modality, the attended stream was cued 200–800 ms *before* the onset of the stimulation train (Giabbiconi et al., 2004, 2007; Adler et al., 2009). We also demonstrated top-down attentional modulation of concomitant nociceptive and visual SS-EPs only when the onsets of the concomitantly presented stimulation trains were shortly delayed to facilitate the selection of the attended stream (Colon et al., 2014). Therefore, the delayed cue in Blöchl and collaborators could have impaired the attentional selection of the attended stream. Moreover, the attentional effect was assessed only during the 2 s of stimulation that followed the cue. This time interval might have been too short for an attentional modulation to be highlighted.

A second important concern relates to the functional significance of the painful SS-EPs (PSS-EPs). As highlighted by the authors, it is not clear whether PSS-EPs actually differed from SS-EPs elicited by innocuous somatosensory input. The scalp topographies of their PSS-EPs were significantly lateralized toward the hemisphere contralateral to the stimulated hand, and resembled closely the scalp topographies of innocuous somatosensory SS-EPs (Giabbiconi et al., 2004, 2007). Most importantly, these scalp topographies were clearly different from the non-lateralized fronto-central scalp topography of SS-EPs elicited by periodic nociceptive stimulation (Mouraux et al., 2011; Colon et al., 2012, 2014). Moreover, the concentric electrode used to elicit PSS-EPs has been suggested to activate selectively nociceptive afferents only when very low stimulation intensities are used (de Tommaso et al., 2011; Perchet et al., 2012; Legrain and Mouraux, 2013). In Blöchl and collaborators, it is likely that the average intensity of 1.03 mA activated a significant proportion of non-nociceptive somatosensory fibers. Consequently, the PSS-EPs could predominantly reflect activity generated by the activation of non-nociceptive somatosensory afferents.

Therefore, although Blöchl and collaborators present an interesting approach to investigate cortical responses evoked by sustained painful stimulation, we believe that critical factors regarding the used methodology and the functional significance of the SS-EPs elicited by intra-epidermal electrical stimulation should be considered prior to concluding that, unlike other sensations, tonic pain is relatively insensitive to top-down modulation by spatial attention. Yet, uncertainty still abounds regarding the exact parameters required to observe attentional modulation of PSS-EPs.

## Author contributions

EC had the initial ideas and wrote the first draft of the manuscript. All authors then revised the manuscript critically and contributed to and have approved the final manuscript.

### Conflict of interest statement

The authors declare that the research was conducted in the absence of any commercial or financial relationships that could be construed as a potential conflict of interest.
